# Wolfram Syndrome presenting with optic atrophy and diabetes mellitus: two case reports

**DOI:** 10.1186/1757-1626-2-9355

**Published:** 2009-12-19

**Authors:** Masoud Reza Manaviat, Maryam Rashidi, Seyed Mohammad Mohammadi

**Affiliations:** 1 Yazd Diabetes Research Center, Shahid Sadoughi University of Medical Science, Jomhouri Boulevard, Yazd, 89179-45556, Iran

## Abstract

Wolfram syndrome is the constellation of juvenile onset diabetes mellitus and optic atrophy, known as DIDMOAD (Diabetes Insipidus, Diabetes Mellitus, Optic Atrophy, and Deafness).

Patients demonstrate diabetes mellitus followed by optic atrophy in the first decade, diabetes insipidus and sensorineural deafness in the second decade, dilated renal outflow tracts early in the third decade, and multiple neurological abnormalities early in the fourth decade.

This study reports two siblings with late diagnosed wolfram syndrome with diabetes insipidus, diabetes mellitus, optic atrophy, deafness and severe urological abnormalities.

In conclusion, cases having early onset insulin-dependent diabetes mellitus and optic atrophy together need to be evaluated with respect to Wolfram.

## Background

For the first time in 1938, wolfram described four siblings with diabetes mellitus and optic atrophy [1]. The main features of wolfram syndrome are diabetes mellitus, diabetes insipidus, sensorineural deafness and optic atrophy [2,3]. It is a progressive neurodegenerative disorder in which patients present with nonautoimmune and non-HLA linked diabetes mellitus associated with optic atrophy in the first decade; diabetes insipidus and sensorineural deafness in the second decade; renal tract abnormalities early in the third decade; and multiple neurological abnormalities, like cerebellar ataxia, myoclonus, and psychiatric illness early in the fourth decade [4]. Wolfram patients usually die from central respiratory failure as a result of brain stem atrophy in their third or fourth decade [4]. The clinical phenotype of wolfram shows similarity with mitochondrial disorders, such as maternally inherited diabetes and deafness, mitochondrial encephalopathy, mitochondrial myopathy, lactic acidosis and stroke-like episodes, or Leber's hereditary optic neuropathy and some studies have concentrated on mitochondrial pathology in Wolfram. Mitochondrial disturbances at the biochemical, morphological, and molecular level have been explained in patients with wolfram, but this has not been a strong finding [5-7].

This study reports two siblings with wolfram and described the course of disease in them to draw the attention of health professionals to think of this syndrome in patients with IDDM and optic atrophy.

## Case presentation

### Case report 1

First case is an Iranian 20-year old female patient admitted to ophthalmology clinic complaining decreased visual acuity. She has suffered progressive visual deterioration since 8 years ago. On fundoscopic examination, bilateral optic atrophy was recognized, and there was no evidence of diabetic retinopathy. Her visual acuity reduced to 20/80 on both sides. Her visual fields demonstrated generalized constriction. IDDM was diagnosed at the age 6, for which she is receiving insulin with rather poor control of her blood sugar. She has a history of nocturia and high urine output (12 liter per day) for 6 years and after that she has had incontinency and since 2 years ago she has had voiding difficulty and has used self intermittent catheterization four times a day. Renal sonography showed pelvicalyceal dilatation in both kidneys equal to hydronephrose grade IV with dilation in proximal of urethra and bladder enlargement secondary to high urine output. Urethral pressure profile shows atonic bladder. Accordingly wolfram was suspected by ophthalmologist and referred her to an endocrinologist. Diabetes insipidus was diagnosed by dehydration test and after starting Minirin drop, urine output decreased and voiding difficulty improved. There was decreased audible acuity by the age of 16 and audiometry showed bilateral high frequency sensorineural hearing loss.

Renal function, hepatic function, thyroid function and other blood test were normal.

Urea: 30 mg/dL, Creatinine: 1 mg/dL, Creatinine clearance = 85 ml/min, Calcium: 10.7 mg/dL, Phosphorus: 4.8 mg/dL, Sodium: 135 mg/dL, Potassium: 4.6 mg/dL, Alanine transaminase (ALT): 40 IU/L, Aspartate transaminase (AST):40 IU/L, Alkaline Phosphatase: 315 U/L, Total Bilirubin: 0.8 mg/dL, Direct Bilirubin: 0.1 mg/dL, T4: 8.4 ng/dL, T3: 120 ng/dL, TSH: 2.5 mU/L, FT4: 1.1 ng/dL.

There was a family history of similar conditions among her brother and one of her cousins. Also she has a 20-year old brother who is normal.

### Case report 2

**Figure 1:**
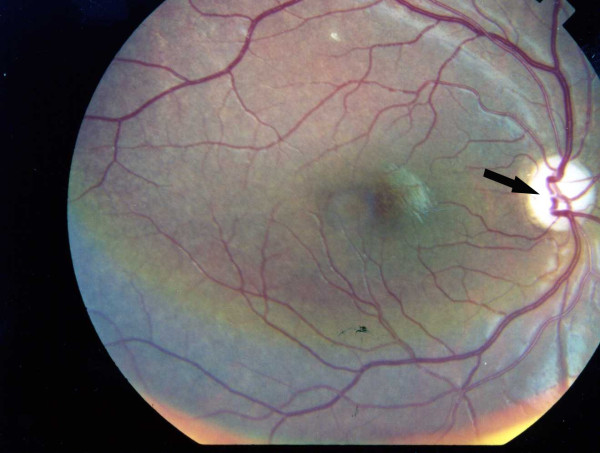
**Photographic image of the patient right eye showing optic atrophy without diabetic retinopathy**.

**Figure 2:**
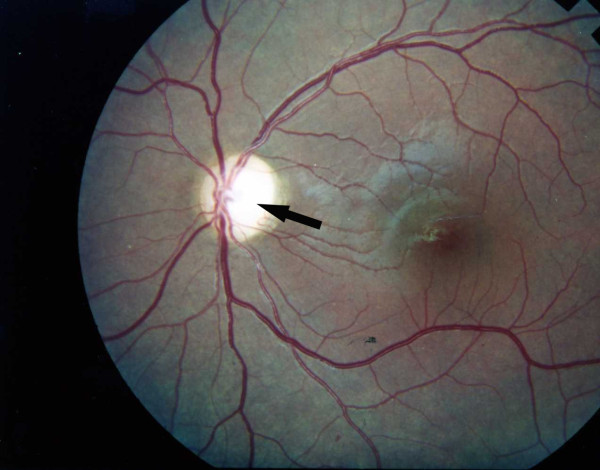
**Photographic image of the patient left eye showing optic atrophy without diabetic retinopathy**.

First case has a 14-year old brother, who acquired IDDM at the same age as his sister, and has had a similar course of disease. He was diagnosed with IDDM at the age of 6 years and has been receiving insulin since then. He has had nocturia, polyuria and voiding difficulty for 3 years and has used self intermittent catheterization too. His visual acuity reduced to 20/120 on both sides. On fundoscopic examination, he has bilateral optic atrophy without diabetic retinopathy (Figure 1, and Figure 2). Renal sonography showed pelvicalyceal dilatation in both kidneys equal to hydronephrose grade III with dilation in proximal of urethra and bladder enlargement. Urethral pressure profile shows atonic bladder. Subsequently wolfram was diagnosed for him and Minirin started. Audiogram was normal. Renal and hepatic function, thyroid function and other blood tests were normal.

## Discussion

Wolfram is a neurodegenerative disease, which incidence is estimated at one in every 770,000 live births [8]. Early-onset IDDM and optic atrophy are believed to be the initial and basic features of the syndrome [9]. Patients usually demonstrate diabetes mellitus associated by optic atrophy in the first decade, sensorineural deafness and diabetes incipidus in the second decade, dilated renal outflow tracts early in the third decade, and several neurological abnormalities early in the fourth decade [10]. The prevalence, severity and age of onset of the various manifestations of this syndrome are not constant in other studies [11]. In our cases diabetes insipidus, optic atrophy and dilated renal outflow tracts were appeared in second decade.

Patients with wolfram demonstrate progressive ophthalmologic symptoms that usually occur after diabetes mellitus. Besides optic atrophy, constriction of visual fields and decline visual acuity and color vision are the other ophthalmological findings in wolfram syndrome [9,12]; however, diabetic retinopathy is rarely observed [10]. Only five cases with Wolfram have been reported without optic atrophy [10]. In our cases, visual acuity of both eyes was 20/80 and 20/120 respectively. Fundus examination revealed optic atrophy, but no sign of diabetic retinopathy was seen. Also, concentric narrowing was present in the field of vision in both cases.

A wide range of urological abnormalities have been observed in Wolfram, including various degrees of upper urinary tract dilatation and bladder dysfunction. An atonic bladder with a large capacity has been mostly reported. Cremers et al. [13] and Barret et al. [4] reported urological abnormalities in 13 and 58% of their patients, respectively, which was seen in our cases as well.

Sensorineural hearing loss or an abnormal audiogram was observed in 39-62% of patients [4,13]. Audiometry showed bilateral high frequency sensorineural hearing loss in one of our cases, but in second case was not seen.

Wolfram is considered to have an autosomal recessive mode of inheritance. Finding from different studies suggest that wolfram is contributed by alternations in genes located on chromosome 4 or alternatively, in the mitochondrial DNA [10]. Since the initial recognition of the WFS 1 gene by Inoue et al. [14], different research has determined more than 50 distinct mutations of this gene. Accordingly, it is postulated that wolfram is genetically heterogenic [10]. We did not perform genetic analyses in our cases; however, our cases' mother, father, and one sibling underwent systemic and ophthalmological examination. There were no manifestations of wolfram in their examinations, but our cases had a cousin with these symptoms that was died from renal disorder.

## Conclusion

In summary, IDDM and optic atrophy are the first and essential features of Wolfram followed by development of deafness, diabetes insipidus and urinary tract abnormalities. Generally cases having IDDM and optic atrophy together need to be evaluated with respect to Wolfram. Cases with Wolfram should be followed all their lives. In these cases, rehabilitation to a normal social life is possible with good follow-up and treatment. The disorder should be kept in mind particularly in our part of the world, where consanguinity is prevalent.

## Consent

Written informed consent was obtained from the patients for publication of this case report and accompanying images. A copy of the written consent is available for review by the Editor-in-Chief of this journal.

## Competing interests

The authors declare that they have no competing interests.

## Authors' contributions

MRM carried out ophthalmologic examination and revised the manuscript. MR helped with data collection and drafted the manuscript. SMM participated in the management of the patients. All authors read and approved the final manuscript.
